# FFAR1/GPR40 Contributes to the Regulation of Striatal Monoamine Releases and Facilitation of Cocaine-Induced Locomotor Activity in Mice

**DOI:** 10.3389/fphar.2021.699026

**Published:** 2021-08-20

**Authors:** Yuko Sadamura, Shanta Thapa, Ryota Mizunuma, Yuki Kambe, Akira Hirasawa, Kazuo Nakamoto, Shogo Tokuyama, Koji Yoshimoto, Kazunori Arita, Atsuro Miyata, Tatsuki Oyoshi, Takashi Kurihara

**Affiliations:** ^1^ Department of Pharmacology, Graduate School of Medical and Dental Sciences, Kagoshima University, Kagoshima, Japan; ^2^ Department of Neurosurgery, Graduate School of Medical and Dental Sciences, Kagoshima University, Kagoshima, Japan; ^3^ Department of Genomic Drug Discovery Science, Graduate School of Pharmaceutical Sciences, Kyoto University, Kyoto, Japan; ^4^ Department of Clinical Pharmacy, School of Pharmaceutical Sciences, Kobe Gakuin University, Hyogo, Japan

**Keywords:** FFAR1, GPR40, serotonin, 5-HT, cocaine, locomotor activity, striatum

## Abstract

The free fatty acid receptor 1 (FFAR1) is suggested to function as a G protein-coupled receptor (GPR40) for medium-to-long-chain free fatty acids. Previous studies on the expression of FFAR1 revealed that the nigrostriatal region is one of the areas which express abundant FFAR1 mRNA/protein in the central nervous system (CNS). However, the role of FFAR1 in the CNS has been still largely unclarified. Here, we examined a possible functional role of FFAR1 in the control of extracellular concentrations of striatal monoamines and cocaine-induced locomotor activity. Microdialysis analysis revealed that the basal level of extracellular dopamine (DA) was significantly elevated, while the basal serotonin (5-HT) level tended to be reduced in the striatum of FFAR1 knockout (−/−) mice. Interestingly, local application of a FFAR1 agonist, GW9508, markedly augmented the striatal 5-HT release in FFAR1 wild-type (+/+) mice, whereas topical application of a FFAR1 antagonist, GW1100, significantly reduced the 5-HT release. However, the enhanced 5-HT release was completely lost in −/− mice. Although acute administration of cocaine enhanced the locomotor activity in both +/+ and −/− mice, the magnitude of the enhancement was significantly reduced in −/− mice. In addition, intraperitoneal injection of GW1100 significantly decreased the cocaine-induced locomotor enhancement. These results suggest that FFAR1 has a facilitatory role in striatal 5-HT release, and the evoked 5-HT release might contribute to enhance cocaine-induced locomotor activity.

## Introduction

The free fatty acid receptor 1 (FFAR1) is a G-protein-coupled receptor (also known as GPR40), which is originally demonstrated to be abundantly expressed in pancreatic β-cells and activated by medium-to-long-chain (C12-C22) free fatty acids (FFAs) (Briscoe et al., 2003; Itoh et al., 2003; Kotarsky et al., 2003; Stoddart et al., 2008; Mancini et al., 2013). Since FFAs have long been recognized as important regulators of glucose homeostasis *via* their ability to stimulate insulin secretion in the presence of glucose, FFAR1 became a promising therapeutic target for type 2 diabetes treatment since its deorphanization, and a number of small-molecule FFAR1 agonists are under development as drugs for type 2 diabetes (Ghislain and Poitot, 2016; Hara, 2016; Kimura et al., 2020).

Previous studies, including our reports, have demonstrated the presence of FFAR1 in the human, primate, and rodent central nervous system (CNS) based on mRNA measurements and immunohistochemical analyses and suggested that FFAR1 plays important physiological roles in the CNS (Briscoe et al., 2003; Ma et al., 2007; Ma et al., 2008; Boneva and Yamashima, 2012; Nakamoto et al., 2012; Nakamoto et al., 2013; Zamarbide et al., 2014; Karki et al., 2015; Nakamoto et al., 2015; Nakamoto et al., 2017). For instance, we have demonstrated that hypothalamic FFAR1 exerts an antinociceptive effect through the facilitated release of endogenous opioid peptides (Nakamoto et al., 2013). In addition, we have further shown that FFAR1 is found to be expressed on descending noradrenergic and serotonergic neurons in the locus coeruleus and rostral ventromedial medulla (RVM), respectively, and the local application of a FFAR1 agonist, GW9508, into these areas evokes a descending endogenous pain control system (Nakamoto et al., 2015; Nakamoto et al., 2017). Furthermore, we have recently found that the disfunction of FFAR1 signaling also contributes to the emotional-related behaviors (Nishinaka et al., 2014; Aizawa et al., 2016; Aizawa et al., 2017; Aizawa et al., 2018) and FFAR1-deficient (FFAR1−/−) mice showed altered monoamine levels in several brain areas including hippocampus, midbrain, hypothalamus, and medulla oblongata (Aizawa et al., 2016). These results suggest that one of the functions of FFAR1 in the CNS might control the central monoaminergic system.

Thus, to further pursue this hypothesis in this study, we have tested whether FFAR1 plays a role in the regulation of striatal monoamine release. Moreover, we analyzed cocaine-induced locomotor stimulation activity in both FFAR1−/− mice and mice treated with a FFAR1 antagonist to evaluate the functional significance of striatal FFAR1, since cocaine is a well-known uptake blocker of the monoamine transporters, and resultant increases in striatal monoamines are important for the cocaine-induced enhanced locomotor responses (Kelly and Iversen, 1976; Joyce et al., 1983; Reith et al., 1997; Cornish and Kalivas, 2001; Han and Gu, 2006; Lüscher and Ungless, 2006).

## Materials and Methods

### Animals

FFAR1−/− mice were generated by homologous recombination (Aizawa et al., 2016; Nakamoto et al., 2017). Briefly, exon 1 of the *Ffar1* gene was replaced with a PKG-neo cassette. Frozen *Ffar1* −/− fertilized oocytes were inoculated into pseudopregnant foster mother (ICR) at Center for Animal Resources and Development, Kumamoto University (CARD ID 1882) and bred at the Institute of Laboratory Animal Science Research Support Center, Kagoshima University. FFAR1−/− mice were backcrossed to C57BL/6J background for >10 generations and maintained by crossbreeding of heterozygous mice. Only male mice were used in this study. To confirm ablation of *Ffar1*, we performed PCR on genomic DNA from tail or ear auricle using the following primers: 40-S1: 5′ TAG GAC TGG CTT CTG GTG CT 3′, 40-AS2: 5′ CCT CCT GAG TTG TGG TGG AT 3′, 3 primer-neo: 5′ GGC TAT TCG GCT ATG ACT GG 3’. This primer pair yielded a 1,166-bp DNA fragment for +/+ mice and a 1,300-bp fragment for −/− mice. Primers were obtained from Integrated DNA Technologies, KK (Tokyo, Japan).

We also employed the quantitative real-time PCR method (see below) to evaluate the presence or absence of FFAR1 in the striatum with +/+ mouse pancreas tissue samples as a positive control. As depicted in Supplementary information (Supplementary Figure S1), the transcript level of FFAR1 was high in the pancreas, but significant expression was also detected in the striatum, where, as expected, the expression was undetectable in FFAR1−/− mice. These results are consistent with our previous immunoblot analysis in the mouse CNS (Nakamoto et al., 2012) and qPCR analysis in the human brain by Briscoe et al. (2003).

Mice were housed under controlled temperature (24 ± 1°C) and humidity (55 ± 10%) with a 12 h light-dark cycle with food and water freely available. All mice used aged 8–22 weeks at the start of each experiment. The animal experiments were approved by the Animal Care Committees of Kagoshima University (approval no. MD16077) and were conducted in accordance with the ethical guidelines for the study of experimental pain in conscious animals of the International Association of the Study of Pain.

### *In Vivo* Microdialysis

The microdialysis experiments were carried out on awake FFAR1+/+ and −/− mice following the protocol described elsewhere (Han et al., 2002). Briefly, mice were anesthetized with pentobarbital sodium (50 mg/kg, i.p.). Microdialysis probes (A-I-(-3)-02 probe, 0.22 o.d., 2.0 mm active membrane length, molecular weight cut-off 50,000 Da, EICOM, Kyoto, Japan) were implanted vertically into the dorsal striatum (anterior, 0.0; lateral, 1.8 mm; and vertical, 4 mm from the bregma) (Carboni et al., 2001; Han et al., 2002). After the surgery, animals were allowed a recovery period of 24 h. On the test day, the probe was connected to a syringe pump (EICOM, Kyoto, Japan) and continuously perfused with Ringer’s solution (in mM: 147 NaCl, 4 KCl, and 2.3 CaCl_2_) at a rate of 2 μl/min. Samples were collected for 50–75 min to determine the basal levels of dopamine (DA), serotonin (5-hydroxytryptamine; 5-HT), and their metabolites, dihydroxyphenylacetic acid (DOPAC), 3-methoxytyramine (3-MT), homovanillic acid (HVA), and 5-hydroxyindoleacetic acid (5-HIAA). Then, GW9508 (a FFAR1 agonist, MW 347.4; 100 μM; Cayman chemical company, Ann Arbor, MI, United States), GW1100 (a FFAR1 antagonist, MW 520.6; 100 μM; Cayman) or cocaine (20 mg/kg; Dainippon Pharmaceutical Co., Ltd, Osaka, Japan) was administered. In the case of examining the effects of the FFAR1 ligands, GW9508 or GW1100 was added to Ringer’s solution perfusing the microdialysis probe for 50 min and samples were collected for another 200 min. While GW9508 was applied to both FFAR1+/+ and −/− mice, GW1100 was only given to +/+ mice. Cocaine was intraperitoneally (i.p.) injected into both +/+ and −/− mice and samples were collected for another 175–200 min. Stock solutions of these FFAR1 ligands (10 mM each) were prepared by dissolving in ≧ 99.9% dimethyl sulfoxide (DMSO; Sigma-Aldrich, St. Louis, MO, United States) and diluted with Ringer’s solution. The concentrations of the FFAR1 ligands were selected based on our preliminary experiments. The stock solution of cocaine (100 mg/ml) was prepared by dissolving in MilliQ water and diluted with saline. The dose of cocaine was chosen based on previous reports (Reith et al., 1997; Han et al., 2002). All samples were automatically injected into the HTEC-500 microdialysis analysis system (Eicom) every 25 min using a fully automatic online system. Concentrations of DA and 5-HT and their metabolites in the microdialysis samples were measured by HPLC with electrochemical detection. The potential of the glassy carbon working electrode (WE-3G, EICOM) was +700 mV vs. the Ag/AgCl reference electrode. The separation was achieved on a 150 × 2.1 (i.d.) mm Eicompak SC-50DS column (EICOM). The mobile phase was a mixture of methanol and 0.1 M citrate–0.1 M sodium acetate buffer (pH 3.9) (17:83, v/v) containing sodium 1-octanesulfonic acid (140 mg/L) and EDTA-2Na (5 mg/L). The flow rate was 0.23 ml/min. Chromatographic data were acquired and processed by the use of an EPC-500 software (Power Chrom, EICOM). The detection limit for DA and 5-HT was estimated to ∼0.05 pg in 50 μl injected onto the column. At the end of the microdialysis experiments, mice were sacrificed, and their brains were removed and correct probe locations were confirmed by visual inspection.

### Open-Field Test

The open-field test was made of polyvinyl chloride plates and was 50 cm×50 cm×40 cm in size. Each FFAR1+/+ and −/− mouse was transferred to the center of the field, and its locomotor activity was measured for 5 min using a color tracking system (CompACT VAS; Muromachi Kikai, Tokyo, Japan) as described previously (Saegusa et al., 2000).

### Cocaine-Induced Locomotor Activity

On the test day, FFAR1+/+ and −/− mice were habituated to the open-field test chamber for 63 min before cocaine (20 mg/kg) injection. The spontaneous locomotion was recorded for 3 min in every 10 min. After i.p. injection of cocaine, locomotor activity was recorded for another 33 min.

In the case of examining the effects of the FFAR1 antagonist (GW1100), vehicle or GW1100 (10 mg/kg) was i.p.-injected into FFAR1+/+ mice after the 63 min habituation, and locomotor activity was similarly measured for 33 min. Then, cocaine (20 mg/kg) was given and activity was monitored for a further 33 min. GW1100 was initially dissolved in ≧ 99.9% DMSO (5 mg/ml) and then diluted with saline (1 mg/ml, final DMSO concentration ∼20%). Thus, ∼ 20% DMSO in saline was employed as vehicle control.

### Quantitative Real-Time PCR

Total RNA was isolated from the striatal tissue including the area *in vivo* microdialysis experiments performed by using Sepasol RNA I Super G reagent (Nacalai Tesque, Kyoto, Japan) and treated with DNase (Promega, Madison, WI) to avoid DNA contamination. cDNA synthesis was carried out using the High-Capacity cDNA Reverse Transcription Kit (Thermo Fisher Scientific) according to manufacturer’s instructions. Quantitative PCR was performed with Thunderbird SYBR qPCR Mix (Toyobo Life Science, Osaka, Japan) using a Thermal Cycler Dice (Real Time System TP800, Takara Bio Inc., Shiga, Japan). Amplification of target cDNA was normalized to *GAPDH* expression. Relative levels of target mRNA expression were calculated using the standard curve method. At least, two independent qPCR experiments of two individual striatum samples were performed. The sequences of primer pairs are described below. Primers were obtained from Integrated DNA Technologies, KK:

FFAR1 (*Ffar1*): 5′ GGG CTT TCC ATT GAA CTT GTT AG 3′ (forward), 5′ GCC CAG ATG GAG AGT GTA GAC C 3′ (reverse).

DA transporter (DAT: *slc6a3*): 5′ ACC ACA CCC GCT GCT GAG TAT T 3′ (forward), 5′ GGT CAT CAA TGC CAC GAC TCT G 3′ (reverse).

5-HT transporter (5HTT: *slc6a4*): 5′ AGG AAC GAA GAC GTG TCC GA 3′ (forward), 5′ CCA AAC CCA GCG TGA TTA ACA T 3′ (reverse).

D1 receptor (*Drd1*): 5′ CGG CCT TAT CGG TCA TAT TGG 3′ (forward), 5′ CTG TGG GTA ACG GGT TGG A 3′ (reverse).

D2 receptor (*Drd2*): 5′ CAG CTC CAA GCG CCG AGT T 3′ (forward), 5′ GGC AGG GTT GGC AAT GAT ACA C 3′ (reverse).

GAPDH: 5′ GAA GGT CGG TGT GAA CGG AT 3′ (forward), 5′ CTC GCT CCT GGA AGA TGG TG 3′ (reverse).

### Western Blot Analysis

Western blot analysis was conducted as previously described (Karki et al., 2015; Ohnou et al., 2016). Mice were deeply anesthetized with sodium pentobarbital (60 mg/kg, i.p.), and dorsal striatal tissues were quickly dissected out. The tissue was homogenized in a lysis buffer [150 mM NaCl, 1 mM EDTA, 1% NP-40, 0.5% sodium deoxycholate, 0.1% SDS, and 50 mM Tris-HCl, pH 8.0] with a mixture of protease and phosphatase inhibitors (Roche Diagnostics, Mannheim, Germany). Protein concentrations were determined with a Bio-Rad protein assay kit (Bio-Rad, Hercules, CA). Proteins (5–10 μg) were separated by SDS-PAGE (12.5% gel) and then transferred to a polyvinylidene difluoride membrane (Millipore). The following antibodies were used: anti-DA transporter monoclonal antibody (rat, 1:200, Millipore MAB369), anti-5-HT transporter polyclonal antibody (rabbit, 1:2,000, Alomone Labs AMT-004), anti-D1 DA receptor monoclonal antibody (rat, 1:100, Sigma-Aldrich D2994), and anti-D2 DA receptor polyclonal antibody (rabbit, 1:2,000, Millipore AB5084P). Immunoreactivity was detected by using an ECL Prime kit (GE Healthcare, Buckinghamshire, United Kingdom). We observed that the anti-DAT and 5HTT antibodies could exhibit single bands with expected molecular weight (∼80 and ∼60 kDa, respectively) in our experimental condition. For the anti-D1 and D2 receptor antibodies, the specificities were validated with D1 and D2 receptor deficient mice (Stojanovic et al., 2017). An anti-β-actin antibody (mouse monoclonal, 1:2,000; Santa Cruz Biotechnology, Inc., Dallas, TX) was used to normalize protein loading. Relative intensities of the bands were quantified by using an image analysis system with ImageJ software, version 1.46 (National Institutes of Health, Bethesda, MD). At least two independent immunoblot experiments of two individual striatum samples were analyzed.

### Statistical Analysis

Experimental data are expressed as mean ± SEM. Single comparisons were made using Student’s two-tailed unpaired *t*-test corrected for unequal variance (Welch’s correction) when appropriate. All remaining data were compared using one-way analysis of variance (ANOVA) with repeated measures followed by Dunnett’s post hoc test or two-way repeated measures ANOVA, with genotype or drug treatment as the between-subject factor and time as the within-subject factor, followed when necessary, by Tukey-Kramer post hoc test. *p* < 0.05 was considered statistically significant.

## Results

### Baseline Levels of Monoamines and Their Metabolites in the Striatum

**FIGURE 1:**
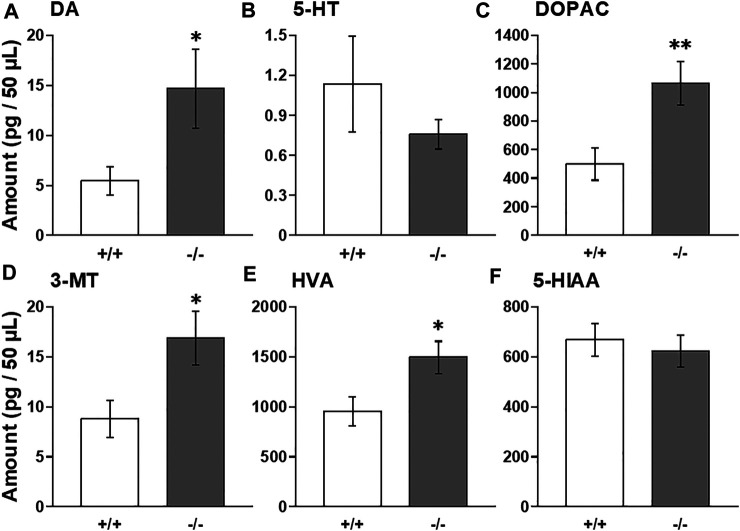
Basal extracellular DA, 5-HT, and their metabolites levels in the striatum in awake FFAR1+/+ and −/− mice. FFAR1 −/− mice showed significantly elevated basal DA level **(A)**. All metabolites of DA, DOPAC **(C)**, 3-MT **(D),** and HVA **(E)** also showed significantly increased levels in −/− mice. Mean baseline 5-HT level **(B)** was substantially reduced in −/− mice, but those of 5-HT metabolite, 5-HIAA **(F)**, did not differ significantly between the two genotypes. *p < 0.05, **p < 0.01 vs. +/+ mice (Student’s *t*-test). *n* = 17 for +/+ mice, and *n* = 16 for −/− mice.

To investigate whether FFAR1 indeed regulates monoamine levels, we employed *in vivo* microdialysis method in this study. At first, we examined baselines levels of extracellular DA, 5-HT and their metabolites within the striatum of awake normal FFAR1+/+ and −/− mice. We found that baseline DA level in the striatum was significantly higher in −/− mice compared with +/+ mice, while a trend for decreased 5-HT baseline levels was observed in −/− mice (Figures 1A,B). The baseline levels of all DA metabolites, DOPAC, 3-MT, and HVA, also showed significant increases in −/− mice, although the level of 5-HT metabolite, 5-HIAA, was not changed (Figures 1C–F).

### FFAR1 Regulated Extracellular 5-HT Level

**FIGURE 2:**
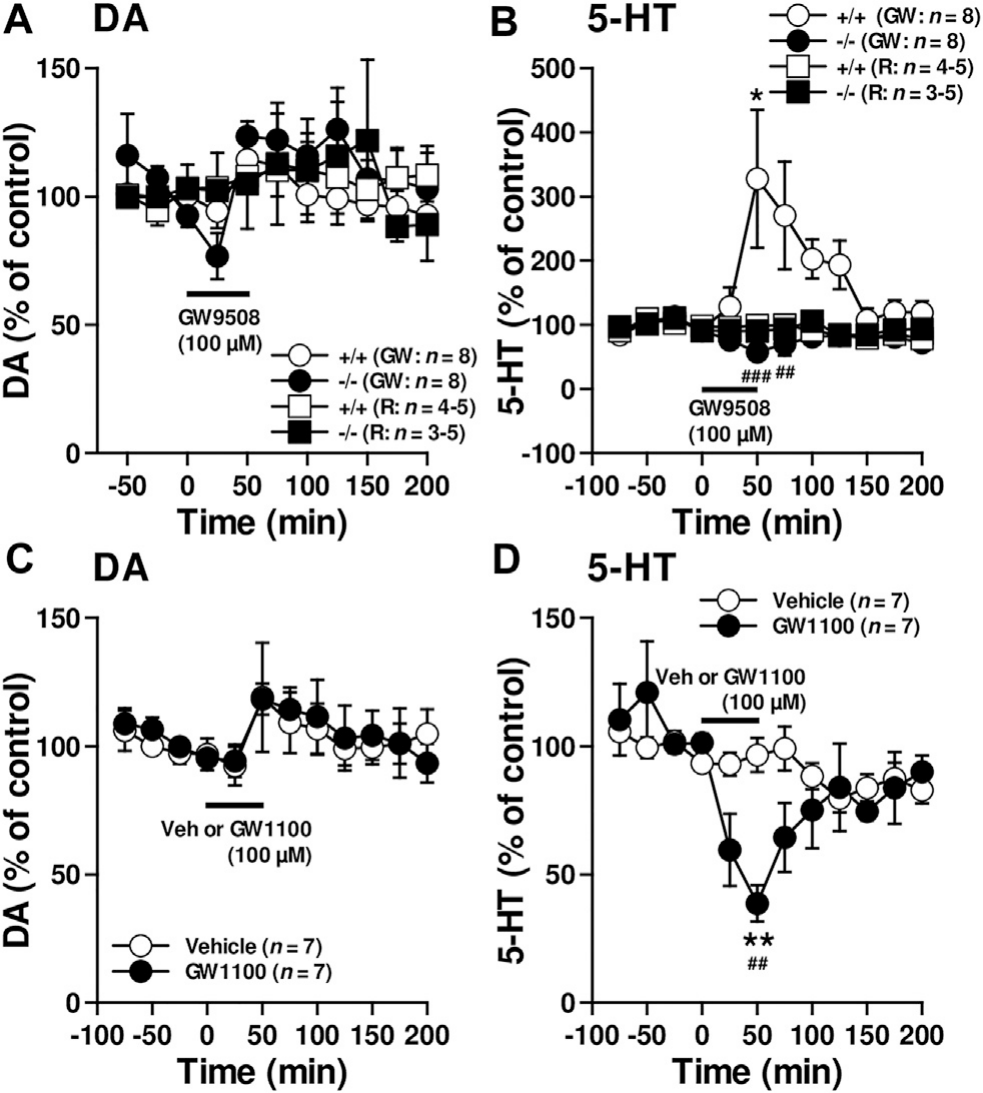
Reciprocal control of striatal 5-HT release by FFAR1 ligands. A FFAR1 agonist, GW9508, increased, but a FFAR1 antagonist, GW1100, decreased striatal 5-HT release without significant changes striatal DA release in FFAR1+/+ mice. However, the facilitatory effect of 5-HT release by GW9508 was completely lost in −/− mice. GW9508 (100 μM) and GW1100 (100 μM) were locally applied through a microdialysis probe as indicated by black lines. We also depicted control data from both +/+ and −/− mice by perfusing Ringer’s solution (R) during all experimental periods **(A,B)**. The figures show mean percentage changes from baseline DA **(A,C)** and 5-HT **(B,D)** values. *p < 0.05, **p < 0.01 versus the basal values at time 0 min (Dunnett’s test). Two-way ANOVA with repeated measures indicated a significant effects of genotype [F_(1, 154)_ = 27.1, *p* < 0.001], time [F_(10, 154)_ = 2.21, *p* < 0.05], and genotype × time [F_(10, 154)_ = 3.93, *p* < 0.01] in the facilitatory influence of GW9508 on 5-HT release **(B)**, and a significant effects of treatment [F_(1, 140)_ = 5.41, *p* < 0.05], time [F_(11, 140)_ = 3.94, *p* < 0.001], and treatment × time [F_(11, 140)_ = 2.90, *p* < 0.01] in the inhibitory action of GW1100 on 5-HT release **(D)**. The post hoc tests showed that GW9508-induced enhanced 5-HT releases were significantly suppressed at 50 and 75 min time points in −/− mice, whereas GW1100 significantly reduced 5-HT release at 50 min time point in +/+ mice. ^##^ *p* < 0.01, ^###^ *p* < 0.001 vs. +/+ mice or vehicle control (Turky-Kramer test).

The *in vivo* microdialysis experiments suggested that FFAR1 in fact directly/indirectly regulated at least striatal DA and 5-HT levels. To evaluate how FFAR1 control striatal DA and 5-HT levels, further *in vivo* microdialysis experiments were performed by *in situ* application of a FFAR1 agonist, GW9508, and an antagonist, GW1100 (Briscoe et al., 2006; Stoddart et al., 2007) (Figure 2). Although we observed a slight transient reduction of DA level in FFAR1−/− mice after local application of GW9508 (100 μM in the perfusing Ringer’s solution) (Figure 2A), GW9508 perfusion resulted in different changes in 5-HT release between FFAR1 +/+ and −/− mice (Figure 2B). In +/+ mice, GW9508 significantly increased the 5-HT level in the striatum. Intriguingly, the stimulated release of 5-HT was almost completely lost in −/− mice. In contrast to what was found in the GW9508 application, local treatment of GW1100 significantly decreased the 5-HT level in +/+ mice (Figure 2D). Despite the downregulation of 5-HT release by GW1100 being remarkable, DA level was not significantly affected by the FFAR1 antagonist (Figure 2C).

### Locomotor Activity in a Novel Environment (5 min Open-Field Test)

**FIGURE 3:**
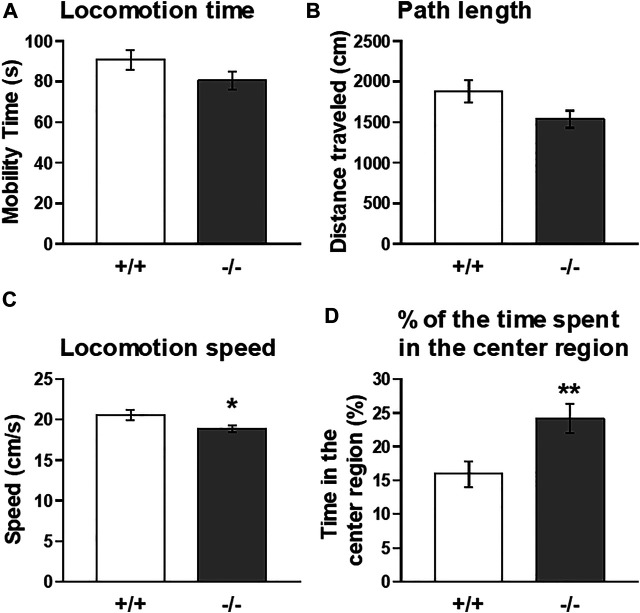
Basal locomotor activity in FFAR1+/+ and −/− mice in response to a novel environment. An open-field test for a total of 5 min showed a slight reduction in locomotion time **(A)** and path length **(B)** in −/− mice. However, significant differences in locomotion speed **(C)** and the percentage of the time spent in the center region **(D)** were noticed in the −/− mice. *p < 0.05, **p < 0.01 vs. +/+ mice (Student’s *t*-test). *n* = 15 per genotype.

Thus, to further explore the functional significance of striatal FFAR1, we employed a cocaine-induced locomotor enhancement model in this study and examined the behavioral phenotype of FFAR1−/− mice. Moreover, we also tested the effects of GW1100 on cocaine-evoked locomotor enhancement. First, we checked baseline locomotor activity under a novel condition (Figure 3). Naïve FFAR1+/+ and −/− mice were placed in a testing chamber (open-field box) and locomotor activity was recorded for 5 min. In accord with our previous report (Aizawa et al., 2016), FFAR1−/− mice spent more time in the center region of the field when compared with +/+ mice (Figure 3D and Supplementary Figure S2), although there was no significant difference between +/+ and −/− mice in the distance traveled (Figure 3B). In this study, we also analyzed additional parameters of the locomotor activity, the locomotion time, and speed and found that locomotion speed was slightly but significantly slower in −/− mice (Figures 3A,C).

### Cocaine-Induced Acute Locomotor Enhancement Was Attenuated in FFAR1−/− and FFAR1 Antagonist-Treated +/+ Mice

Then, we next studied the functional role of FFAR1 by examining locomotor activity in response to acute cocaine injections in both FFAR1+/+ and −/− mice. Locomotor stimulation is one of the most characteristic effects of psychostimulants including cocaine (Kelly and Iversen, 1976; Joyce et al., 1983; Reith et al., 1997; Cornish and Kalivas, 2001; Han and Gu, 2006; Lüscher and Ungless, 2006).

**FIGURE 4:**
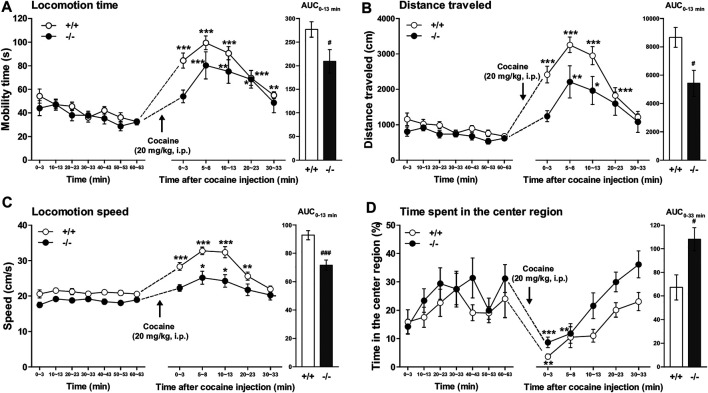
FFAR1−/− mice showed reduced cocaine-induced locomotor responses. Mice were placed in an open-field test chamber for 63 min for habituation purpose (left panels in **A–D**). Then, cocaine was i.p.-injected, and mice were returned to the test chamber for an additional 33 min (middle panels in **A–D**). Cocaine injection (20 mg/kg, i.p.) induced significant acute enhancement of locomotor activity in both FFAR1+/+ and −/− mice. *p < 0.05, **p < 0.01, ***p < 0.001 versus the basal (pre-cocaine injection) values (60–63 min time points) (Dunnett’s test). However, FFAR1−/− mice showed reduced cocaine-evoked acute locomotor responses analyzed in terms of the three parameters (right panels in **A–C**), but −/− mice showed enhanced ambulation in the center region after cocaine injection (right panel in **D**). Two-way ANOVA with repeated measures indicated significant effects of genotype [F_(1, 116)_ = 9.32, *p* < 0.01 **(A)**, F_(1, 116)_ = 15.9, *p* < 0.001 **(B)**, F_(1, 116)_ = 43, *p* < 0.001 **(C)**, F_(1, 116)_ = 12.1, *p* < 0.001 **(D)**] and time [F_(5, 116)_ = 18.7, *p* < 0.001 **(A)**, F_(5, 116)_ = 17.7, *p* < 0.001 **(B)**, F_(5, 116)_ = 43, *p* < 0.001 **(C)**, F_(5, 116)_ = 11.8, *p* < 0.001 **(D)**]. Since significant interactions between genotype and time were not detected in **A–D**, each area under the curve (AUC) value, calculated for 13 min **(A–C)** and 33 min **(D)** after acute cocaine injection, was compared between +/+ and −/− mice. ^#^ *p* < 0.05, ^###^ *p* < 0.001 vs. +/+ mice (Student’s *t*-test). +/+: *n* = 9–11 for +/+ mice, and *n* = 11 for −/− mice.

Both FFAR1+/+ and −/− mice underwent a 63 min habituation in a testing open-field box prior to cocaine (20 mg/kg) injection. Except for locomotion speed, there were no significant differences in the other three parameters (locomotion time, distance traveled, and time spent in the center region) during the habituation period (Figure 4 and Supplementary Figure S3). An acute administration of cocaine significantly increased locomotor activities in both +/+ and −/− mice. However, the overall locomotor responses to cocaine in terms of three parameters (locomotion time, distance traveled, and locomotion speed) were significantly reduced in −/− mice relative to +/+ mice (Figures 4A–C). Interestingly, we observed a change in the pattern of horizontal locomotion. Although we could not detect a significantly increased percentage of the time spent in the center region in −/− mice before cocaine injection with this experimental condition (3 min measurement in every 10 min), we could detect significantly increased ambulation of −/− mice in the center area of the open-field chamber after cocaine injection (Figure 4D).

**FIGURE 5:**
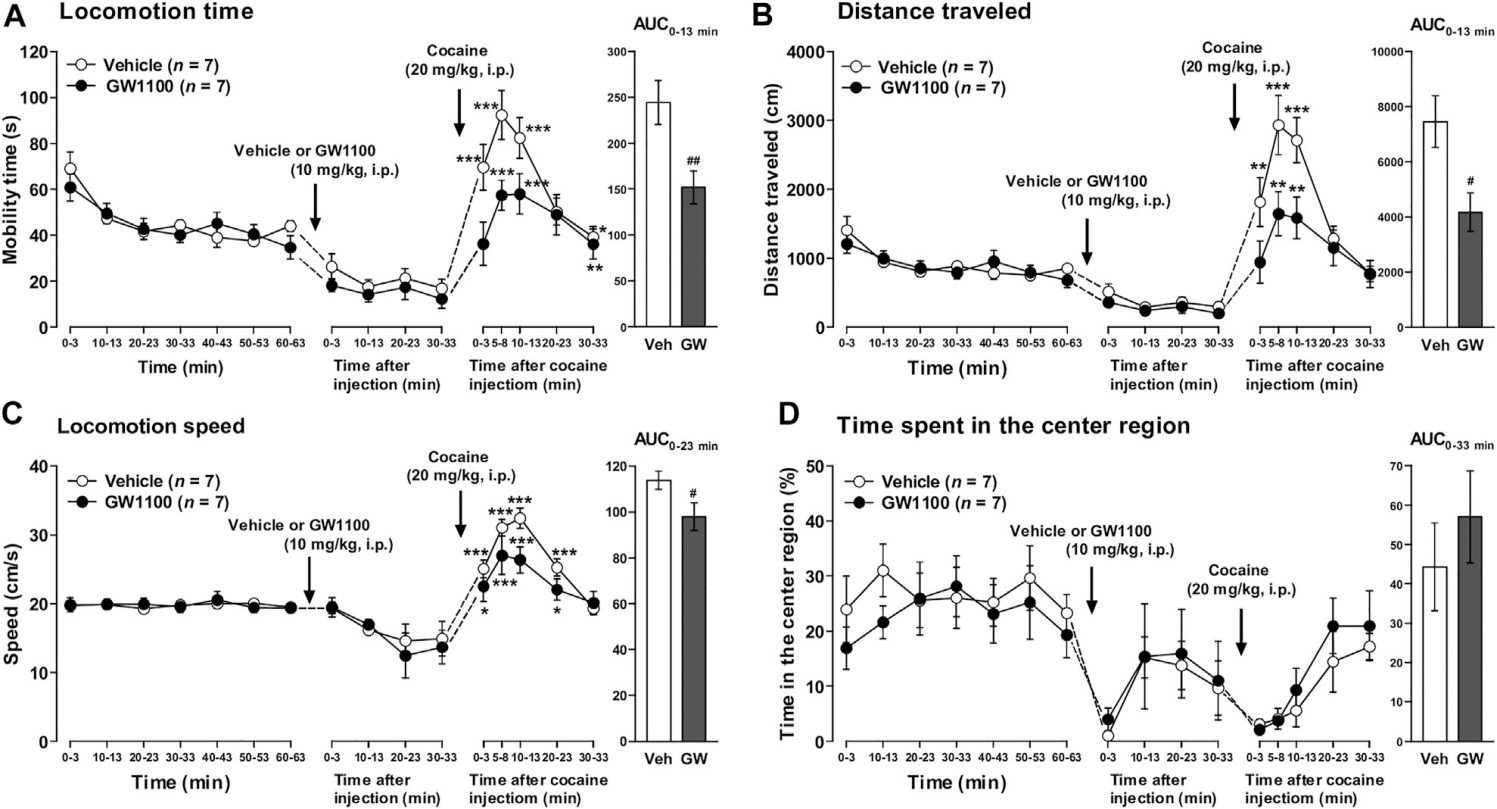
GW1100 (a FFAR1 antagonist) reduced cocaine-induced acute locomotor responses in FFAR1+/+ mice. Mice were placed in an open-field test chamber for 63 min for habituation purpose (leftmost panels in **A–D**). Next, GW1100 (10 mg/kg) or vehicle (∼20% DMSO in saline) was i.p.-injected, and mice were placed in the open-field test chamber for another 33 min (second from the left panels in **A–D**). Then, cocaine (20 mg/kg) was i.p.-injected, and mice were returned to the test chamber for an additional 33 min (second from the right panels in **A–D**). Cocaine injection induced significant acute enhancement of locomotor activity in both vehicle and GW1100-treated mice. *p < 0.05, **p < 0.01, ***p < 0.001 versus the basal (pre-cocaine injection) values (Dunnett’s test). However, GW1100 significantly reduced cocaine-induced acute locomotor responses analyzed in terms of the three parameters (rightmost panels in **A–C**), but GW1100 only slightly enhanced ambulation in the center region after cocaine injection (rightmost panel in **D**). Two-way ANOVA with repeated measures indicated significant effects of treatment [F_(1, 72)_ = 14.6, *p* < 0.001 **(A)**, F_(1, 72)_ = 14.3, *p* < 0.001 **(B)**, F_(1, 72)_ = 6.44, *p* < 0.05 **(C)**] and time [F_(5, 72)_ = 16.6, *p* < 0.001 **(A)**, F_(5, 72)_ = 17.1, *p* < 0.001 **(B)**, F_(5, 72)_ = 19.1, *p* < 0.001 **(C)**]. Since significant interactions between treatment and time were not detected in **A–C**, each area under the curve (AUC) value, calculated for 13 min **(A,B)**, 23 min **(C)** and 33 min (**D**: just for comparison) after acute cocaine injection, was compared between vehicle control and GW1100-treated mice. ^#^ *p* < 0.05, ^##^ *p* < 0.01 vs. vehicle control mice (Student’s *t*-test). *n* = 7 each.

As shown in Figure 5, pretreatment of FFAR1+/+ mice with GW1100 (10 mg/kg, i.p.) for 30 min also significantly reduced cocaine-induced enhancement of mobility time, distance travelled, and locomotion speed (Figures 5A–C). Although we could not detect a significant increase in the time spent in the center region, there was a tendency toward an increment of this parameter (Figure 5D).

### Cocaine-Induced Increases in Extracellular DA and 5-HT Levels Were Apparently Normal in FFAR1−/− Mice

**FIGURE 6:**
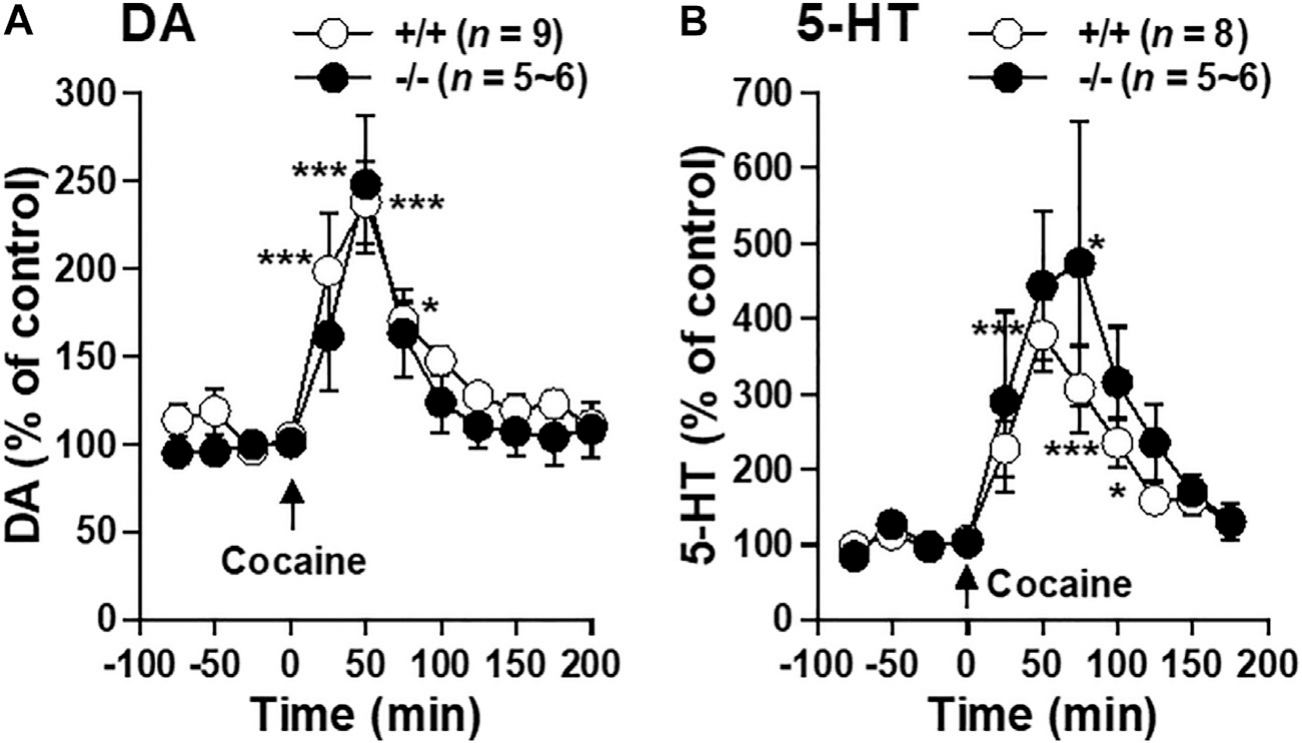
Cocaine-evoked DA and 5-HT releases in the striatum of FFAR1+/+ and −/− mice. At time 0 min. a single i.p. injection of cocaine (20 mg/kg) was given. The figures show mean percentage changes from baseline DA **(A)** and 5-HT **(B)** values. *p < 0.05, ***p < 0.001 versus the basal values at time 0 min of the same genotype (one-way ANOVA followed by Dunnett’s post hoc test).

Since impaired cocaine action on striatal monoamine transmission could be responsible for the blunted locomotor responses seen in FFAR1−/− mice, we examined the ability of acute systemic cocaine injection to elevate extracellular DA and 5-HT using *in vivo* microdialysis approach. As shown in Figures 6A,B, cocaine induced similar magnitude of potentiation in striatal DA and 5-HT levels in both +/+ and −/− mice, suggesting that the function of DA and 5-HT transporters appeared to be normal in −/− mice.

### Expression Levels of Monoamine Transporters and DA Receptors in the Striatum

**FIGURE 7:**
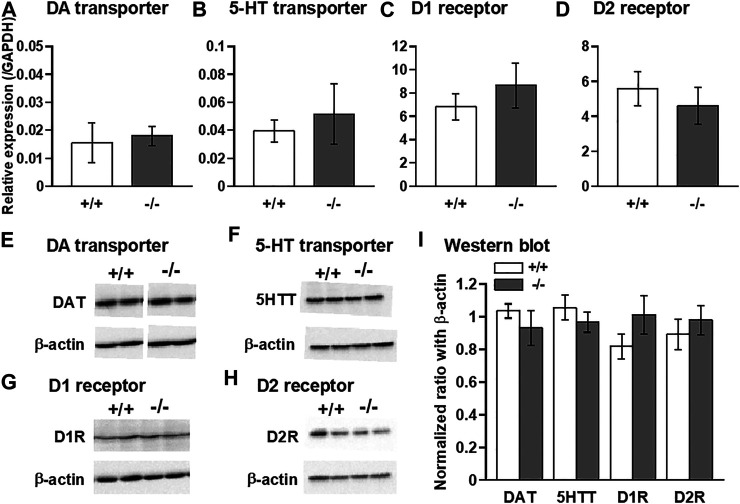
Quantitative PCR and western blot analyses of DA and 5-HT transporters, and dopamine D1 and D2 receptors in the striatum. qPCR **(A–D)** and western blot **(E–I)** analyses showing that no significant differences in the expression levels of DA transporter (DAT: **A,E,I**), 5-HT transporter (5HTT: **B,F,I**), D1 receptor (D1R: **C,G,I**), and D2 receptor (D2R: **D,H,I**) were observed between FFAR1+/+ and −/− mice. *n* = 6 **(A)** and 5 **(B-D)** each. In **(I)**, *n* = 4 (+/+) and 6 (−/−) for DAT, *n* = 8.

Finally, we examined the expression levels of DA and 5-HT transporters as well as D1 and D2 DA receptors using qPCR and western blot methods. Comparisons of the levels of these mRNAs and proteins between normal +/+ and −/− striata showed no significant differences (Figure 7).

## Discussion

The main findings of the current study include the following: 1) FFAR1 tonically regulated striatal 5-HT release: activation of FFAR1 facilitated 5-HT release, but inhibition of this receptor downregulated the release, and 2) FFAR1−/− mice and mice treated with the FFAR1 antagonist (GW1100) demonstrated reduced locomotor activity in response to cocaine administration. To our knowledge, this study provides the first direct *in vivo* evidence for FFAR1 as an important regulator of striatal 5-HT level. Although further extensive studies are necessary to delineate the mechanism underlying the reduced locomotor responses to cocaine, we could speculate that the facilitatory role of FFAR1 in the striatal 5-HT release would be likely to contribute to the enhancement of acute cocaine-induced locomotor activity.

We found that FFAR1−/− mice displayed a significant increase in the basal level of extracellular DA, as well as its metabolites, with a trend for the decreased basal level of 5-HT. Thus, the FFAs-FFAR1 system was suggested to play an important role in striatal DA and 5-HT effluxes. In fact, previous studies using animals deficient in n-3 polyunsaturated FFAs reported that the deficiency enhanced anxiety-like behaviors in mice (Harauma and Moriguchi, 2011) and modified striatal DA availability or basal DA release in rats (Zimmer et al., 2000; Bondi et al., 2014), which may be at least partly consistent with both our previous (Aizawa et al., 2019) and present data. Conversely, n-3 FFAs, such as eicosapentaenoic acid and docosahexaenoic acid, were suggested to reduce stress and anxiety-like behaviors in animal studies (Song et al., 2007; Aizawa et al., 2019), and supplementation of n-3 FFAs reduced the risk of progression to psychotic disorder and psychiatric morbidity (Amminger et al., 2015). Moreover, our group recently demonstrated that repeated intracerebroventricular administration of GW9508 reduced the duration of immobility behavior in the forced swim test, suggesting that activation of brain FFAR1 may downregulate depression-related behavior (Nishinaka et al., 2014). However, it may be worth noting here that FFAR1−/− mice spent more time in the center region compared with +/+ mice in this open-field test (performed at Kagoshima University; Figure 3D), which concurs with our previous report performed at Kobe Gakuin University (Aizawa et al., 2016). Center versus surround occupancy and locomotion in open-field chambers is a measure of anxiety-like behavior and is sensitive to anxiolytic treatments (Prut and Belzung, 2003), although the interpretation of center/surround measure as an anxiety measure has also been a matter of debate due to the failure of effectiveness in several mouse strains (particularly C57BL/6J strain) to prototypical anxiolytic drugs (Thompson et al., 2015).

The increase in basal DA and its metabolite levels observed in the naïve FFAR1−/− mice might reflect activation of DA neurons such as the increase in DA release, synthesis, and metabolism, and decrease in DA reuptake. However, we could speculate that the striatal DA reuptake activity would be apparently normal, since expression analysis of DA transporter suggested no alterations in FFAR1−/− mice, and cocaine increased DA levels to a similar extent as in +/+ mice (see below). However, further investigations are necessary to determine DA reuptake activity more precisely in −/− mice striatum.

The most important finding in this study is that activation of FFAR1 facilitated 5-HT release and inhibition of this receptor reduced the release in the striatum. Our previous study has demonstrated that FFAR1 colocalized with NeuN (a neuron marker) positive cells and also with tryptophan hydroxylase (a serotonergic neuron marker) positive cells in the RVM (Nakamoto et al., 2015). Thus, it would be possible that axon terminals derived from FFAR1-positive RVM serotonergic neurons contributed to the 5-HT release in the striatum. However, since subcellular localization of FFAR1 in the striatum has not been fully elucidated yet, anatomical data such as whether midbrain DA neurons are under the control of FFAR1-positive serotonergic neurons would be required in future experiments.

It has been known that the central 5-HT system is associated with the modulation (both inhibitory and excitatory) of DA neuronal activity, and both basal and psychostimulant-induced locomotor activity (see below). The extensive body of literature demonstrates the involvement of at least two members of 5-HT_2_ receptor family, 5-HT_2A_ receptor and 5-HT_2C_ receptor, in the regulation of DA release (Higgins et al., 2013; Cunningham and Anastasio, 2014; Howell and Cunningham, 2015). There is a general consensus that 5-HT_2A_ receptor activation stimulates but 5-HT_2C_ receptor activation inhibits DA release. For instance, although 5-HT_2A_ receptor stimulation clearly facilitates DA release in the mesocorticolimbic system in areas key for addiction, 5-HT_2A_ receptor antagonists have no effect on basal DA release in these areas, suggesting that the 5-HT_2A_ receptor is involved primarily in the control of phasic, not tonic, DA release.

Meanwhile, administration of 5-HT_2C_ receptor agonists decreased, whereas 5-HT_2C_ receptor antagonist and inverse agonists increased basal firing rates of the ventral tegmental area (VTA) DA neurons and subsequent DA release within the nucleus accumbens (NAc). A later study found that 5-HT_2C_ receptor knockout mice had increased basal DA levels in the NAc and dorsal striatum, which correlated with the increased tonic activity in the nigral neurons (Abdallah et al., 2009). Thus, these considerations suggest that endogenous striatal FFAR1-evoked 5-HT release might control basal (tonic) DA release through 5-HT_2C_ receptors.

Another important finding of this study is that FFAR1−/− mice and mice i.p.-administered with the FFAR1 antagonist (GW1100) displayed a significantly reduced locomotor response to acute cocaine. It is well documented that increases in extracellular DA levels in the striatum are related to the psychomotor-stimulating effects of cocaine and other psychostimulants (see Introduction). This suggests that altered striatal DA responsivity may change the locomotion in these mice. However, contrary to our expectations, we found in the present study that FFAR1−/− mice also showed a similar magnitude of potentiation by cocaine in DA and 5-HT levels in the striatum. These data might rule out impaired activities of DA and 5-HT transporters in FFAR1−/− mice. Furthermore, qPCR and western blot analysis suggested that the expression levels of DA and 5-HT transporters, as well as those of D1 and D2 receptors, were not significantly changed in the striatum of FFAR1−/− mice. Although a further rigorous study is necessary for possible involvement of FFAR1 signaling system in cocaine-evoked responses, we could present one hypothesis that further elevation of extracellular DA in −/− mice whose baseline DA level was significantly increased compared to +/+ mice might produce impairment of downstream DA receptor signaling mechanisms and lead to attenuated cocaine-induced locomotor enhancement. Thus, we could postulate that one essential role of the striatal FFAR1-5-HT signaling system might be to adjust DA level within an appropriate range.

Recent preclinical studies have clearly indicated that 5-HT plays an important role in the abuse-related effects of cocaine (Cunningham and Anastasio, 2014). Although we could not exclude the involvement of other 5-HT receptor subtypes (see Filip et al., 2010, for review), several lines of evidence suggested that 5-HT_2A_ and 5-HT_2C_ receptors again may be potential targets to treat some aspects of cocaine abuse (Higgins et al., 2013; Howell and Cunningham, 2015). For instance, systemic administration of 5-HT_2A_ receptor antagonists block (McMahon and Cunningham, 2001; Fletcher et al., 2002; Filip et al., 2004) whereas a 5-HT_2A_ receptor preferential agonist DOI (2,5-dimethoxy-4-iodoamphetamine) and virally mediated overexpression of 5-HT_2A_ receptors in the VTA enhance the locomotor-stimulant effects of cocaine (Filip et al., 2004; Herin et al., 2013). Conversely, studies in rodents have reliably found that 5-HT_2C_ receptor agonists attenuate (Grottick et al., 2000; Filip et al., 2004), whereas 5-HT_2C_ receptor antagonists enhance the behavioral effects of cocaine (Fletcher et al., 2002, Fletcher et al., 2006). Thus, although further research is still needed to evaluate the possible involvement of the 5-HT receptor system in the FFAR1-5-HT signaling, we currently hypothesize that 5-HT receptor activation evoked by FFAR1-induced 5-HT release would control the magnitude of cocaine-induced hyperlocomotion.

In conclusion, the present study suggests that FFAR1 plays an important role in 5-HT release and appears to exert substantial modulatory influence on DA level in the mouse striatum. Although future studies are necessary to elucidate the precise roles of FFAR1 in cocaine action including the locomotor responses, this study would promote a better understanding of the neurophysiological function of FFAR1 as a potential therapeutic target of cocaine abuse.

## Data Availability

The original contributions presented in the study are included in the article/Supplementary Materials; further inquiries can be directed to the corresponding author.
