# Supplementary material for: FFAR1/GPR40 Contributes to the Regulation of Striatal Monoamine Releases and Facilitation of Cocaine-Induced Locomotor Activity in Mice
Source: Front Pharmacol. 2021 Aug 20;12:699026. doi: 10.3389/fphar.2021.699026 (PMC8417570; doi:10.3389/fphar.2021.699026)
Supplement: Supplementary file 1 [file DataSheet1.doc]

**Supplementary Figure 1 |** FFAR1 mRNA expression in the striatum and pancreas of normal FFAR1+/+ and -/- mice. FFAR1 mRNA was not detected in -/- striatum. N.D.: not detected.


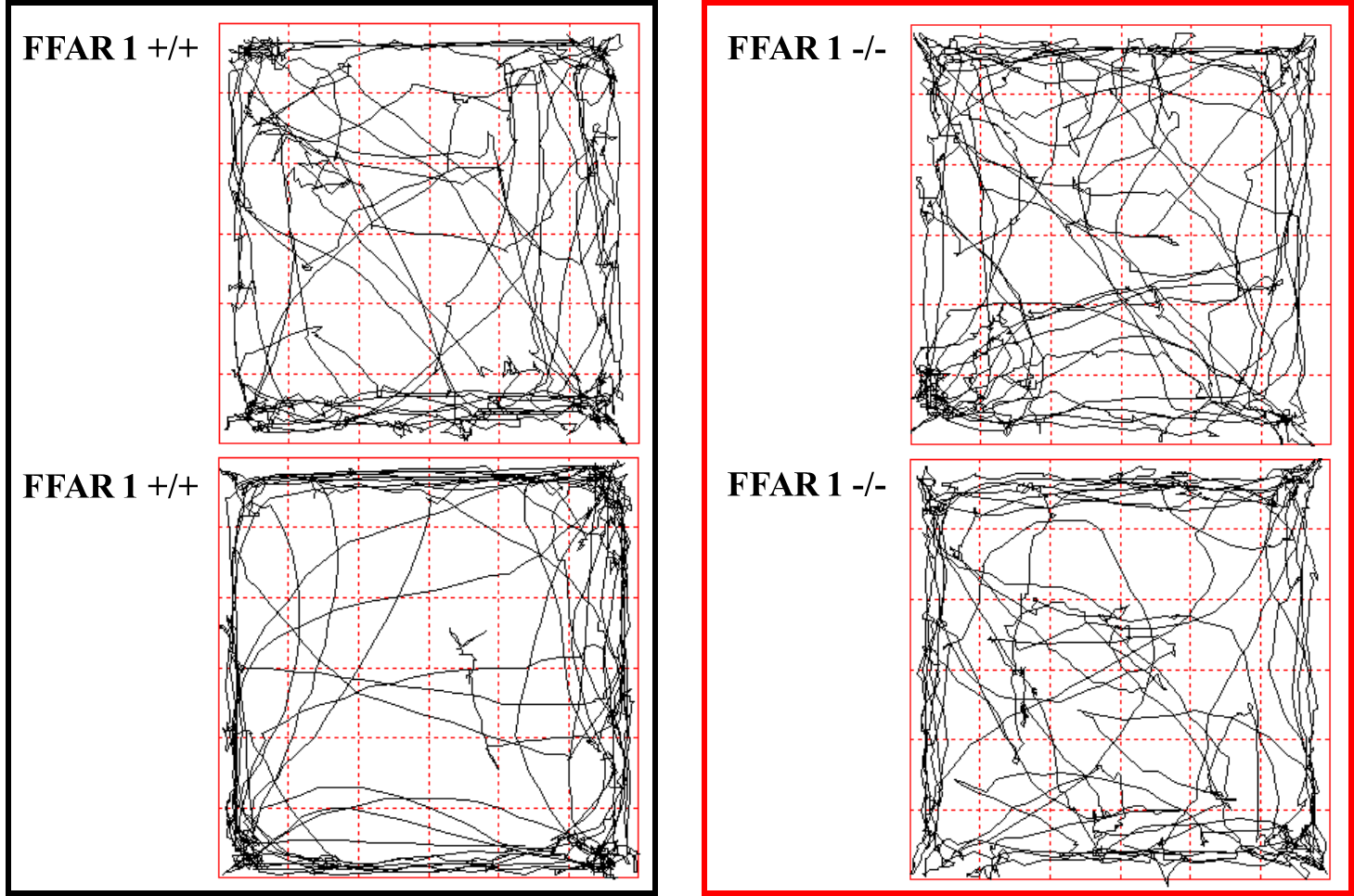


**Supplementary Figure 2 |** Representative examples of walking paths of FFAR1+/+ (Left pannel) and -/- mice (Right pannel) in open-fields test. The center was defined as the inner 16 squares.


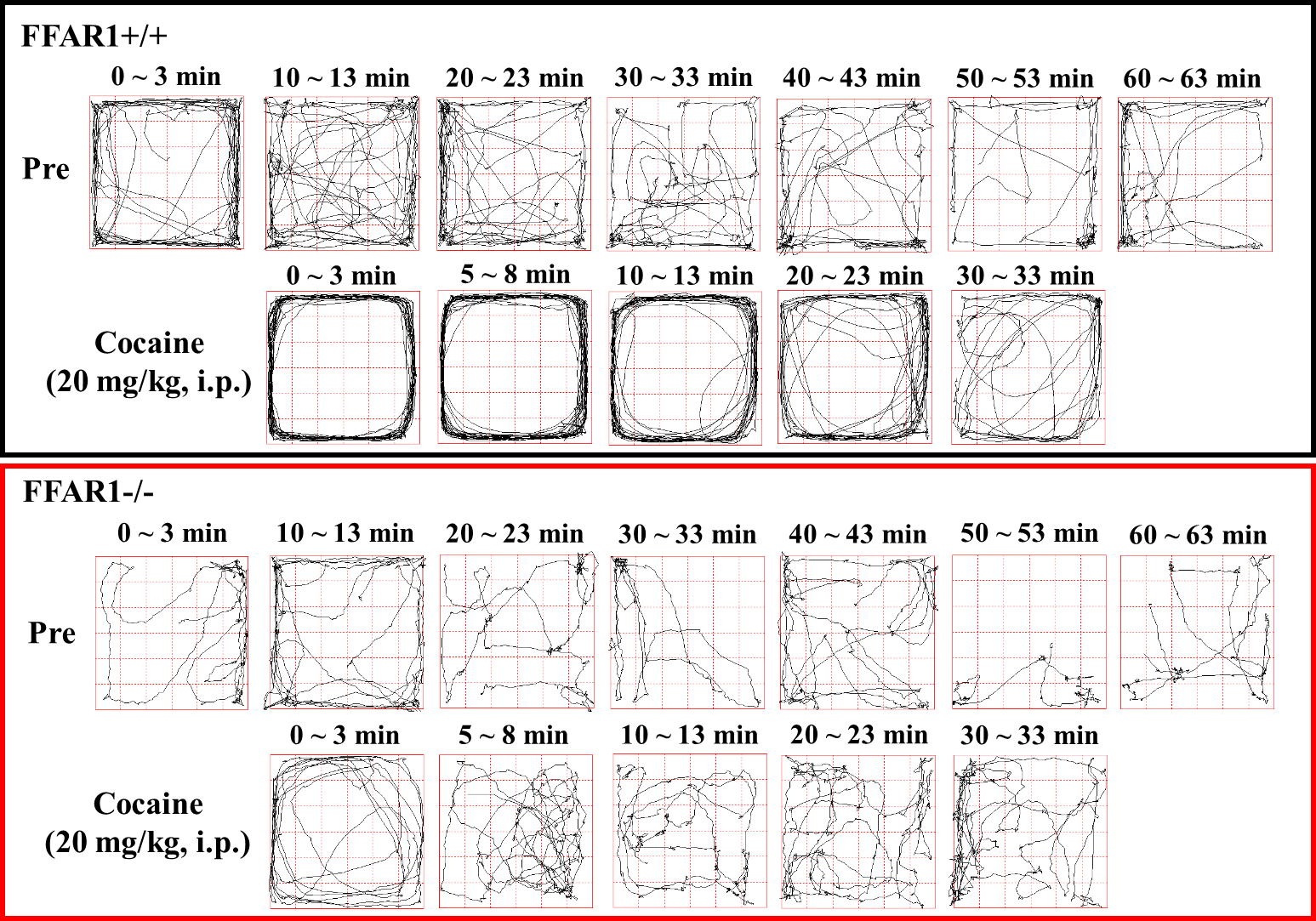


**Supplementary Figure 3 |** Examples of cocaine-induced locomotor activity in FFAR1+/+ and -/- mice. Mice were placed in an open-field test chamber for 63 min for habituation purpose (upper open-field behavioral tracks in each panel). And then, cocaine was i.p.-injected, and mice were returned to the test chamber for an additional 33 min. The spontaneous locomotion was recorded for 3 min in every 10 min.
